# A New Method of the Visualization of the Double-Stranded Mitochondrial and Nuclear DNA

**DOI:** 10.1371/journal.pone.0066864

**Published:** 2013-06-18

**Authors:** Anna Ligasová, Dmytro Strunin, Karel Koberna

**Affiliations:** Laboratory of Experimental Medicine, Institute of Molecular and Translational Medicine, Faculty of Medicine, Palacký University in Olomouc, Olomouc, Czech Republic; University of Toronto, Canada

## Abstract

The study describes the method of a sensitive detection of double-stranded DNA molecules *in situ*. It is based on the oxidative attack on the deoxyribose moiety by copper(I) in the presence of oxygen. We have shown previously that the oxidative attack leads to the formation of frequent gaps in DNA. Here we have demonstrated that the gaps can be utilized as the origins for an efficient synthesis of complementary labeled strands by DNA polymerase I and that such enzymatic detection of the double-stranded DNA is a sensitive approach enabling *in-situ* detection of both the nuclear and mitochondrial genomes in formaldehyde-fixed human cells.

## Introduction

Presently, many approaches are used for the visualization of DNA molecules *in situ*. In this respect, substances that bind to DNA and concurrently serve as fluorescent markers are most frequently used for the labeling of double-stranded DNA. These include substances that bind to the minor groove of DNA, for example DAPI or Hoechst dyes [1], [2], or substances that intercalate in the double-stranded DNA, such as YOYO-1 or TOTO-1 [3], [4]. However, most of these dyes also exhibit an affinity to RNA [3], [5]. This property is therefore a fundamental problem in the detection of the double-stranded DNA in cells which contain a high amount of various RNA molecules. In addition, they usually provide low signal intensity when used for the detection of the mitochondrial DNA in the fixed cells of some species. A typical example are human cells. To overcome these problems, several alternative methods have been developed. They include a relatively time-consuming approach based on *in-situ* hybridization [6], immunocytochemical detection by anti-dsDNA antibodies [7] or detection of incorporated 5-bromo-2′-deoxyuridine (BrdU) by anti-BrdU antibodies [8], [9]. The last two methods suffer from the fact that the relatively weak mitochondrial signal is frequently obscured by a signal emanating from the nuclear DNA. Although it is also possible to use an alternative method based on 5-ethynyl-2′-deoxyuridine, which is usually used for the detection of newly synthesized nuclear DNA [10], it seems that the sensitivity of the microscopic detection of the mitochondrial replication by EdU is lower than by BrdU. Unlike BrdU, EdU obligatorily requires an amplification step [11]–[13].

We describe here a new approach for the visualization of the cellular DNA. It is based on the DNA scission by a radical-oxidative reaction on the carbon atoms of the deoxyribose followed by a set of elimination reactions including the elimination of the nucleobases, finally resulting in the cleavage of the internucleotide linkages and the formation of gaps [8], [14]. Previously, we have shown that the gaps can be utilized for the detection of BrdU in the newly replicated DNA [8], [15]. In the present study, we have used the gaps as the origins for a synthesis of labeled DNA strands. Our results have shown that depending on the conditions used, the nuclear or mitochondrial genome can be visualized preferentially.

## Materials and Methods

### Cell culture, fixation and permeabilization protocol

Human HeLa cells (a generous gift from Dr. David Staněk, Institute of Molecular Genetics, Prague) were cultured on coverslips in a Petri dish in Dulbecco's modified Eagle's medium with L-glutamine (DMEM, Gibco) supplemented with 10% fetal calf serum (PAA Laboratories), 1% gentamicin and 0.85 g/l of NaHCO_3_ at 37°C in a humidified atmosphere containing 5% CO_2_.

The cells were fixed with 2% formaldehyde for 10 minutes, washed in 1×PBS, permeabilized in 0.2% Triton X-100 for 10 minutes and washed in 1×PBS.

### The cleavage of cellular DNA by copper(I) ions

The fixed and permeabilized cells were briefly washed three times in 1×PBS buffer and subsequently incubated in a cleavage solution consisting of 10 mM sodium ascorbate and 4 mM copper(II) sulfate (RT) on a laboratory shaker for either 10 or 30 seconds or 1 or 10 minutes. Alternatively, the solution of 10 mM tetrakis(acetonitrile)copper(I) hexafluorophosphate was applied for the same periods of time. After that, the cells were washed in 20 mM EGTA (30 minutes, RT) on the shaker.

### The enzymes used

These enzymes and conditions were used:

1. Terminal deoxynucleotidyl transferase (TdT; 2 U/µl, 10 minutes, 37°C, Fermentas), a buffer for TdT, 0.05 mM dATP, dGTP, dCTP and alternatively 0.05 mM Alexa Fluor® 555-aha-2′-deoxyuridine-5′-triphosphate (Alexa 555-dUTP), biotin-16-2′-deoxyuridine-5′-triphosphate (biotin-dUTP) or digoxigenin-11-2′-deoxyuridine-5′-triphosphate (digoxigenin-dUTP).
2. DNA polymerase I (0.2 U/µl, 20 minutes, RT, Fermentas), a buffer for DNA polymerase I, 0.05 mM dATP, dGTP, dCTP and alternatively 0.05 mM Alexa 555-dUTP, biotin-dUTP or digoxigenin-dUTP; when biotin-dUTP and digoxigenin-dUTP were used, we also added dTTP or 5-bromo-2′-deoxyuridine-5′-triphosphate (BrdUTP). We found that the addition of 0.05 mM dTTP or BrdUTP resulted in the highest signal. In the case of Alexa 555-dUTP, no such positive effect was observed. Instead, the addition of dTTP or BrdUTP led to a signal decrease.
3. Shrimp alkaline phosphomonoesterase (phosphatase, SAP; 1 U/µl, 20 minutes, 37°C, Fermentas), a buffer for SAP.

### Antibodies

These primary antibodies were used:

Rabbit anti-biotin antibody (1∶100, Enzo), mouse anti-digoxigenin antibody (1∶100, Roche), mouse anti-mitochondrial MTC02 antibody (1∶100, Abcam), mouse anti-BrdU antibody (1∶20, Roche).

These secondary antibodies were used:

Alexa Fluor® 488 anti-mouse antibody or Cy3 anti-mouse antibody and DyLight 649 anti-rabbit antibody (all: 1∶100, Jackson Immunoresearch).

All antibodies were diluted in 1×PBS if not stated otherwise. The cells were incubated with primary and secondary antibodies for 30 minutes at RT. After washing, the cells were mounted in Mowiol and inspected by means of fluorescence microscopy.

### Alternative procedures

The DNA was stained by DAPI (0.3 µM, 20 minutes, RT) or Hoechst 33242 (2 µg/ml, 20 minutes, RT).

### Image acquisition, processing and analysis

The images from fluorescence microscopy were obtained by means of an Olympus IX81 microscope (Olympus) equipped with a Hamamatsu ORCA II camera with a resolution of 1344×1024 pixels using Cell∧R acquisition software. The following objectives were used: LUCPLFLN 20×NA 0.45, UPLFLN oil 40×NA 1.3 and UPLSAPO oil 100×NA 1.4.

For image processing, Adobe Photoshop software was used. For drawing the scheme, Corel Draw software was used.

The mean signal intensity in the nucleus and mitochondria was calculated as the ratio of integral nuclear or mitochondrial intensity and the number of evaluated pixels, respectively. The mean background intensity was calculated as the ratio of the pixel intensity in cytoplasmic regions in the vicinity of the measured mitochondria and the total number of pixels. The signal from 100 cells was evaluated in every experiment.

## Results

We have previously shown that the incubation of formaldehyde-fixed and Triton X100-permeabilized cells with copper(I) ions leads to the formation of frequent gaps in DNA [8]. Here, we use this approach for the detection of nuclear and mitochondrial DNA. We have tested two basic cleavage solutions for the introduction of the gaps in DNA. The first one was based on the use of a mixture of sodium ascorbate and copper(II) sulfate, the second one on a freshly prepared solution of tetrakis(acetonitrile)copper(I) hexafluorophosphate. In both cases, the fixed and permeabilized cells were briefly washed three times in 1×PBS buffer and subsequently incubated in a cleavage solution consisting of either 10 mM sodium ascorbate and 4 mM copper(II) sulfate or the solution of 10 mM tetrakis(acetonitrile)copper(I) hexafluorophosphate. The samples were incubated at RT on a laboratory shaker for either 10 or 30 seconds or 1 or 10 minutes. After that, the cells were washed in 20 mM EGTA (30 minutes, RT) on the shaker. As the presence of oxygen is necessary for a successful DNA cleavage [8], the shaking of the samples during their incubation in the cleavage solution was an essential step. In this respect, already mild mixing was sufficient for an efficient oxygen supply and the subsequent DNA cleavage.

**Figure 1:**
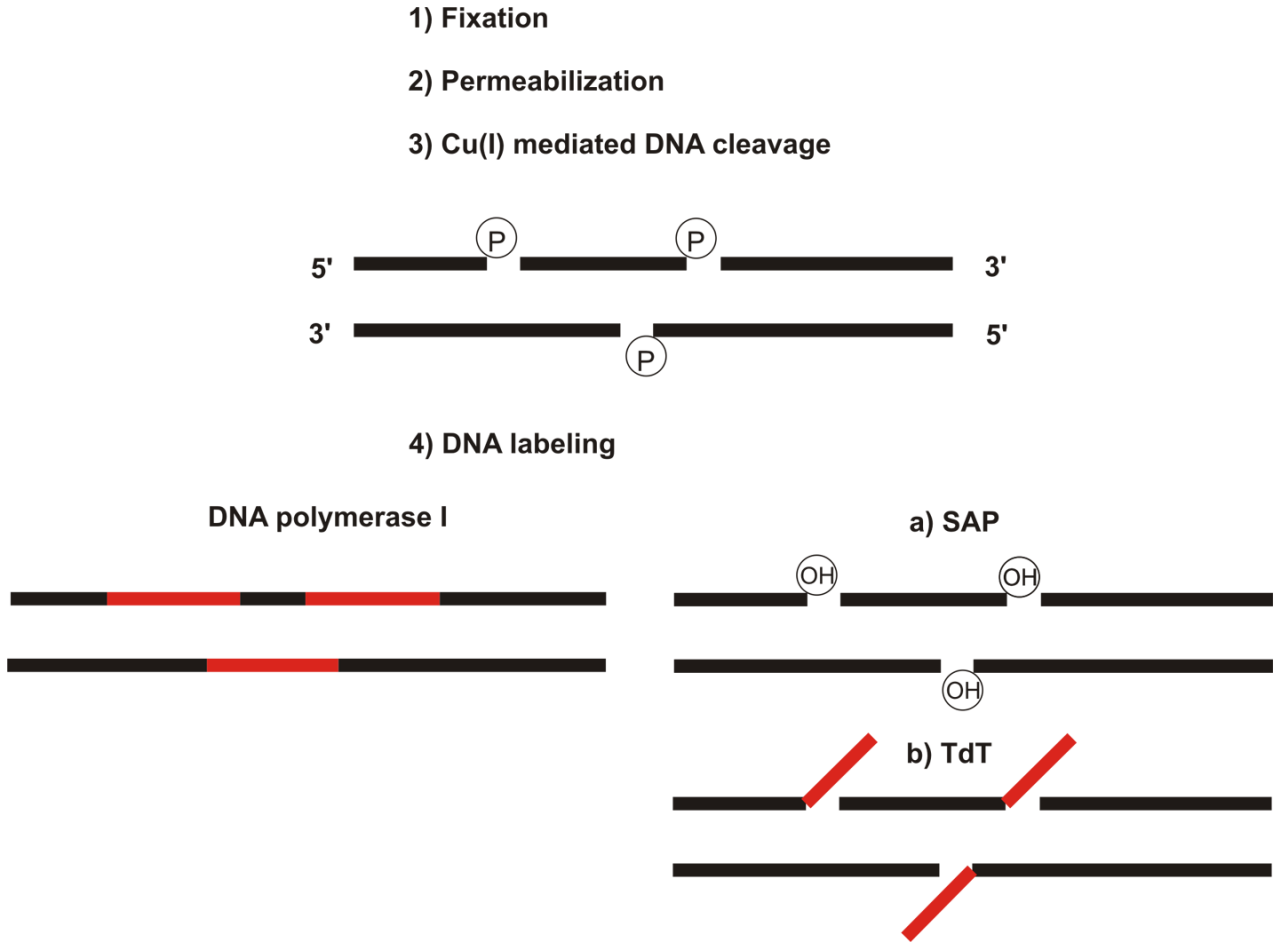
The scheme of the method. A simplified scheme of two basic alternatives of labeling cellular double-stranded DNA is shown. The common steps (fixation, permeabilization and copper(I)-mediated DNA cleavage leading to the gap formation) are followed by the labeling of DNA by means of DNA polymerase I or TdT. In the case of TdT, it is necessary to use the pre-incubation step with SAP in order to reconstitute the hydroxyl groups at the 3′ ends of the gaps. P and OH designate phosphate and hydroxyl groups at the 3′ ends of the gaps, respectively.

**Figure 2:**
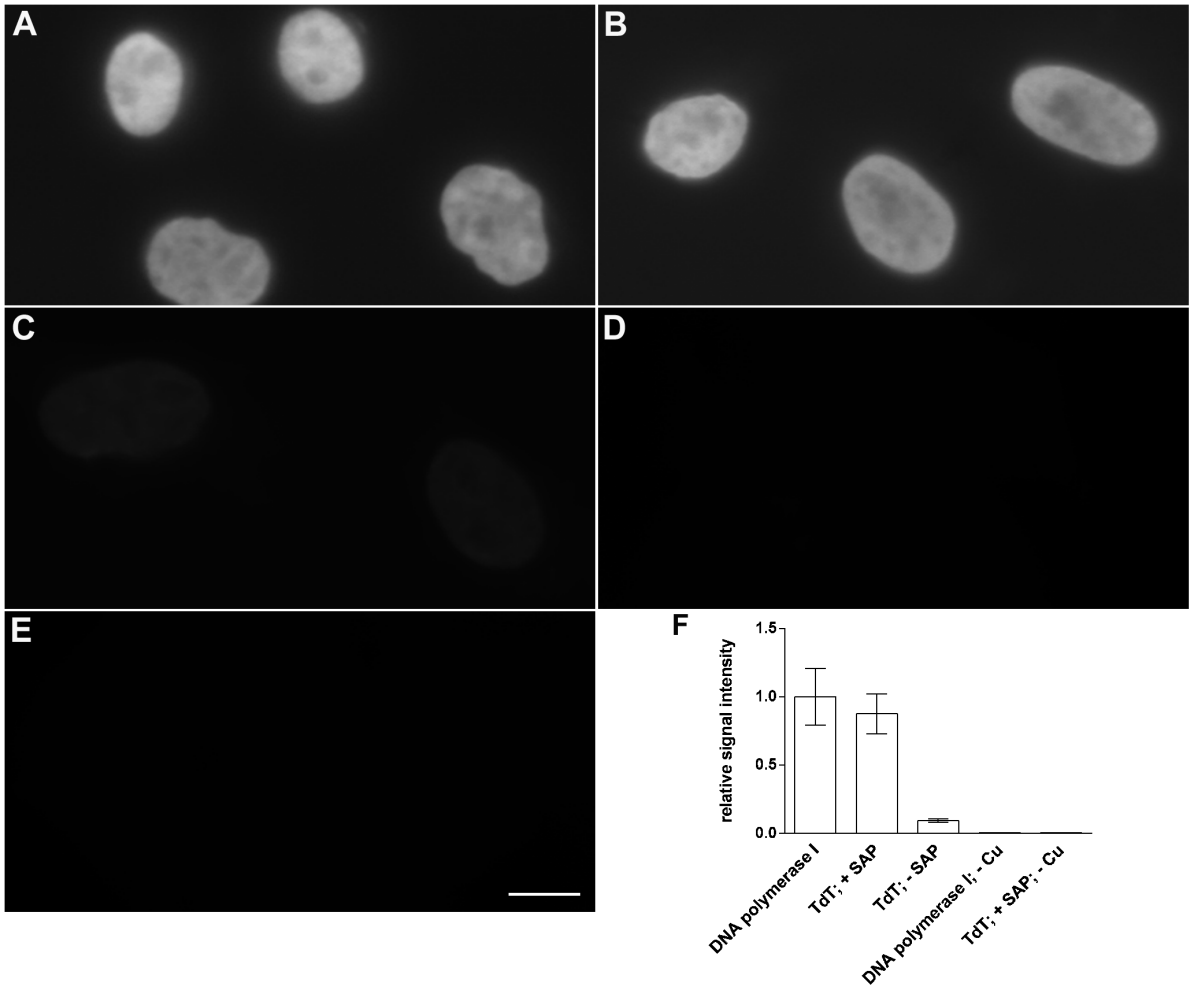
The detection of nuclear DNA in HeLa cells. The detection of nuclear DNA by DNA polymerase I (A, D) or TdT (B, C, E) and Alexa 555-dUTP after 10-minute incubation in the cleavage solution is shown. In the case of TdT, the incubation with TdT was (B) or was not (C) preceded by SAP treatment to remove the phosphate groups from the 3′ ends. The cells were (A, B, C) or were not (D, E) incubated in the cleavage solution for 10 minutes before the enzymatic detection of DNA. The relative mean signal intensities and the standard deviations are shown in (F) for all these experiments. Barr: 20 µm.

After the introduction of the gaps, we used DNA polymerase I or terminal deoxynucleotidyl transferase (TdT) and a mixture of deoxynucleotide triphosphates with Alexa Fluor® 555-aha-2′-deoxyuridine-5′-triphosphate (Alexa 555-dUTP) or biotin-16-2′-deoxyuridine-5′-triphosphate (biotin-dUTP) or digoxigenin-11-2′-deoxyuridine-5′-triphosphate (digoxigenin-dUTP; for the exact composition of the buffers, see the Materials and Methods) for their labeling. Biotin-dUTP was detected in two consecutive steps using the rabbit anti-biotin antibody and anti-rabbit secondary antibody conjugated with DyLight 649. The detection of digoxigenin-dUTP was performed similarly, using primary mouse anti-digoxigenin antibody and anti-mouse antibody conjugated with Alexa Fluor® 488 or Cy3. The schema of the action of both enzymes at the 3′ ends of the gaps is shown in Figure 1. Note that the action of TdT does not require the presence of DNA matrices for the synthesis of labeled DNA strands and can theoretically result in a higher signal than the use of DNA polymerase I. On the other hand, as the phosphate groups are present at the 3′ ends of the gaps created [8], TdT requires a pre-incubation step with shrimp alkaline phosphomonoesterase (SAP). SAP treatment was not necessary for the DNA polymerase I action as the DNA polymerase I possesses an intrinsic 3′ 5′ exonuclease activity and can therefore resume a 3′ hydroxyl group. 3′ 5′ exonuclease activity is absent in the case of TdT. The results of this experiment are shown in Figure 2. They clearly confirm that the DNA polymerase I (Figure 2A) does not require any additional step for its activity and that the assay based on the use of TdT (Figure 2B, C) requires a pre-incubation step with SAP. No signal was observed when the incubation in the cleavage solution was omitted (Figure 2D, E). The relative mean signal intensity in the cell nucleus is shown in the graph in Figure 2F.

As TdT required the action of SAP and provided the same or lower signal than DNA polymerase I under the conditions used, we further tested the protocols based on the use of DNA polymerase I exclusively. Our experiments have also shown that both cleavage solutions provided similar results. However, as the sodium ascorbate and copper(II) sulfate can be prepared as stock solutions and can be kept in this form for long time in the freezer and the refrigerator, respectively, while the solution of tetrakis(acetonitrile)copper(I) hexafluorophosphate has to be prepared fresh before its application, we used solution based on the sodium ascorbate and copper(II) sulfate in the next experiments.

**Figure 3:**
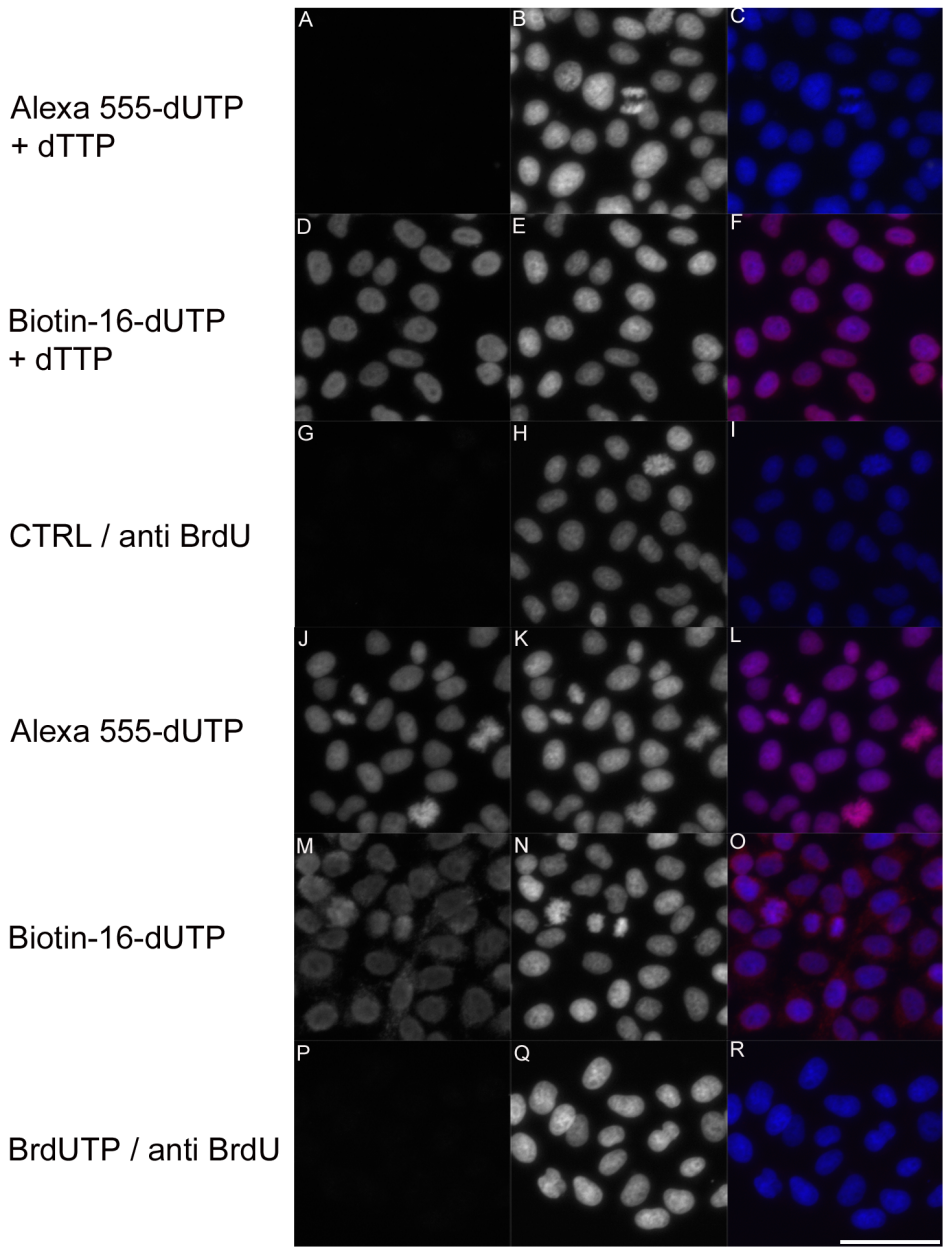
The detection of overall DNA by various marker nucleosides. The detection of overall DNA by DNA polymerase I and either by Alexa 555-dUTP (A, C, J, L; red in the color images) or biotin-dUTP (D, F, M, O; red in the color images) or BrdUTP (P, R; red in the color images) in HeLa cells after 10-minute incubation in the cleavage solution is shown. The cells were simultaneously stained by DAPI (B, C, E, F, H, I, K, L, N, O, Q, R; blue in the color images). The cells in A–F were incubated in the DNA polymerase I mixture containing dTTP. The cells in J–R were incubated in the DNA polymerase I mixture without the addition of dTTP. The cells in G–I were fixed, permeabilized, incubated with anti-BrdU antibody and secondary antibody, and the cells were stained by DAPI. Bar: 50 µm.

We compared the efficiency of the visualization of cellular DNA by Alexa 555-dUTP, biotin-dUTP and digoxigenin-dUTP after 10-minute incubation in the cleavage solution. In these experiments, the cells were incubated in the reaction mixtures with or without the addition of dTTP (see the Materials and Methods). The results of the detection of DNA by Alexa 555-dUTP and biotin dUTP are shown in Figure 3. We found that in the case of Alexa 555-dUTP the addition of dTTP resulted in a signal decrease (compare Figure 3A–C and 3J–L). On the other hand, when biotin-dUTP was used, the highest signal was provided if a mixture containing dTTP was used (compare Figure 3D–F and 3M–O). Similar results were obtained using digoxigenin-dUTP instead of biotin-dUTP (data not shown). Our data also showed that the signal provided by Alexa 555-dUTP resembled the DAPI signal (Figure 3J–L) more faithfully than biotin-dUTP (Figure 3D–F, M–O) or digoxigenin-dUTP (data not shown). In this respect, the biotin-dUTP and digoxigenin-dUTP signals were more homogeneous and less structured than the DAPI signal. In some experiments, we used BrdUTP for labeling the double-stranded DNA; nevertheless, as BrdU is accessible for the reaction with anti-BrdU antibodies exclusively in RNA × DNA strands but not in DNA × DNA strands [16], we did not observe any specific signal in this case (Figure 3P–R). Although the omission of the signal could result from the inability to incorporate BrdUTP into the growing DNA strand, our results do not support this possibility. In this respect, the addition of BrdUTP to biotin-dUTP or digoxigenin-dUTP exhibited the same effect as the addition of dTTP (data not shown). No signal was observed in control sample (Figure 3G–I). In this case, the cells were only fixed with formaldehyde, permeabilized and stained with primary anti-BrdU antibody and secondary antibody and DAPI.

**Figure 4:**
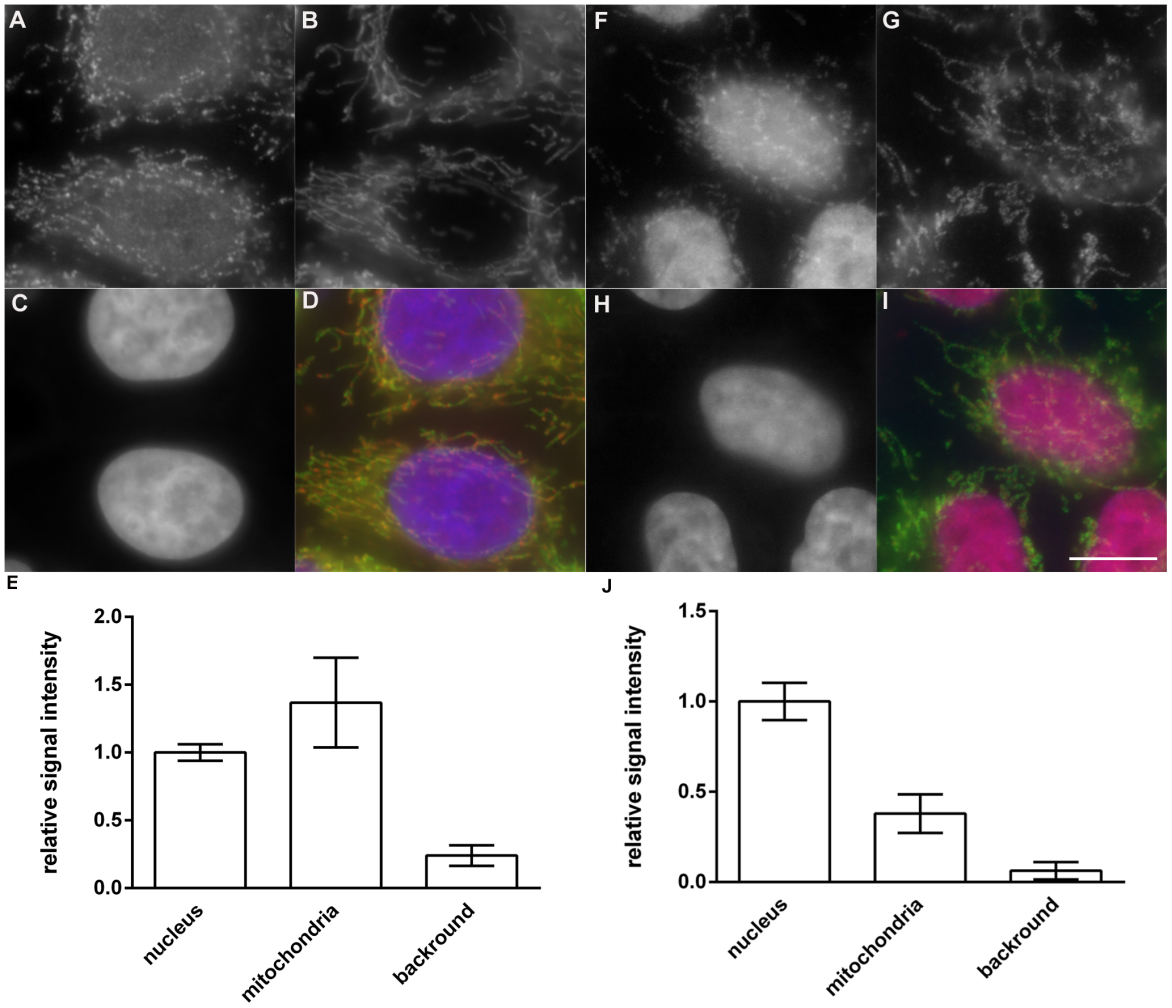
The detection of the mitochondrial genome. The detection of the mitochondrial DNA by a 10-second cleavage in a solution containing copper(I) ions followed by the labeling of mitochondrial DNA by DNA polymerase I and biotin-dUTP (A, D; red in the color image) or Alexa 555-dUTP (F, I; red in the color image). The anti-mitochondrial antibody (B, D, G, I; green in the color images) was used for the identification of mitochondria. DNA was stained by DAPI (C, D, H, I; blue in the color images). The graphs show the relative mean signal intensities and the standard deviations of Alexa 555-dUTP (E) and biotin-dUTP (J) derived signal measured in the nucleus, mitochondria and cytoplasm (background). Bar: 10 µm.

A detailed inspection of the images revealed also a cytoplasmic signal. This signal was undetectable using DAPI or Hoechst stains both in copper(I)-treated and non-treated cells. However, its intensity was relatively weak in comparison with the signal emanating from the nuclear DNA. Moreover, the differences were visible between the cells labeled by Alexa 555-dUTP and the cells labeled by biotin-dUTP and digoxigenin-dUTP. Generally, the ratio between the cytoplasmic signal and the nuclear signal was higher in the case of biotin-dUTP and digoxigenin-dUTP than in the case of Alexa 555-dUTP. We found that the progressive lowering of the incubation time with the copper(I) ions to 10–30 seconds resulted in the reduction of the nuclear signal with a low effect on the cytoplasmic signal. Colocalization studies with anti-mitochondrial antibody have clearly shown that the cytoplasmic signal corresponds to the mitochondrial genome both for the method based on the biotin-dUTP (Figure 4A–E) and Alexa 555-dUTP (Figure 4F–J). The relative mean signal intensity in the mitochondria, cell nucleus and the relative mean intensity of the background is shown in the graphs in Figure 4E and 4J. The obtained data have confirmed that biotin-dUTP (Figure 4E) and digoxigenin-dUTP (data not shown) provides a much higher ratio of the mitochondrial and nuclear signals (M/N ration) than Alexa 555-dUTP (compare Figures 4E and 4J). The M/N ratio was 1.368±0.415 and 0.379±0.145 for biotin-dUTP and Alexa 555-dUTP, respectively. This ratio is clearly of primary importance as the high nuclear signal can completely obscure the mitochondrial signal (compare Figure 2 to Figures 4A and 4F). In this respect, already 1-minute cleavage results in M/N ratio of 0.149±0.0416 for Alexa 555-dUTP. Definitely, these results have also strongly indicated that the enzymatic labeling of the partially-cleaved DNA by copper(I) provides a very efficient method of the detection of mitochondrial DNA. The only prerequisite for the effective and reliable resolution of mitochondrial genomes by wide-field microscopy is the lowering of the nuclear signal that otherwise masks the mitochondrial signal.

## Discussion

We present here a new approach for the detection of double-stranded DNA. It is based on the previously described findings that copper(I) ions in the presence of oxygen produce frequent gaps in the DNA by oxidative attacks on the deoxyribose moiety [8]. The main advantage of the approach presented is the absolute selectivity of the double-stranded DNA, the insensitivity to the presence of RNA and the possibility to reveal the mitochondrial genome specifically.

We tested Alexa 555-dUTP, biotin-dUTP and digoxigenin-dUTP as the markers of the double-stranded DNA. We found that the use of the Alexa 555-dUTP is favorable especially in the cases of the detection of nuclear DNA. On the other hand, the use of biotin-dUTP or digoxigenin-dUTP is much more convenient in the case of the detection of the mitochondrial genome. This difference can be a result of the different geometry of these molecules, because different linkers were used to bind biotin and digoxigenin. We can only speculate that markers linked like those used in the Alexa 555-dUTP molecule will provide the best results for the detection of a nuclear signal and the markers linked like biotin or digoxigenin will provide the best results for the detection of the mitochondrial genome. The superiority of biotin-dUTP and digoxigenin-dUTP for the detection of the mitochondrial genome and Alexa 555-dUTP for nuclear DNA is not clear although it can also be caused by the more relaxed form of the mitochondrial genome than of nuclear DNA. In this respect, nuclear DNA consists of a large number of various proteins including histones in several orders of folding. No such extensive folding is typical for the mitochondrial genome. The more relaxed mitochondrial genome can therefore accept better such spatially less convenient markers as biotin-dUTP or digoxigenin-dUTP. In this respect, both biotin-dUTP and digoxigenin-dUTP required an addition of dTTP for an efficient synthesis of the labeled strands of DNA. No such requirements were observed in the case of Alexa 555-dUTP.

Taking all our results together, the following protocol provided the best result for the specific labeling of mitochondrial/nuclear DNA: The fixation of the cells for 10 minutes in 2% formaldehyde in PBS, a brief wash in PBS, permeabilization in 0.2% Triton X100 in PBS for 10 minutes, a brief wash in PBS, cleavage in the freshly prepared mixture of 4 mM copper sulfate and 10 mM sodium ascorbate on a laboratory shaker for 10 seconds (mitochondrial genome) or 10 minutes (nuclear genome), incubation in 20 mM EDTA on the shaker for 30 minutes, a wash in a DNA polymerase I buffer and incubation in the DNA polymerase I buffer containing dATP, dGTP, dCTP and Alexa 555-dUTP (nuclear genome) or in the DNA polymerase I buffer supplemented with dATP, dGTP, dCTP and dTTP and biotin-dUTP or digoxigenin-dUTP (mitochondrial genome) for 20 minutes at RT.

Besides DNA polymerase I, another useful alternative for the detection of nuclear DNA can be labeling the DNA by TdT. Although TdT requires phosphatase activity for its action as copper(I) treatment results in the formation of phosphate groups on the 3′ends of the gaps [8], its use can be preferable in the cases where the sensitivity of the method is of primary importance as TdT action is not limited by the presence of free DNA matrices. In fact, in the case of the detection of nuclear DNA by means of DNA polymerase I, we did not have any need to improve the method's sensitivity. The application of TdT for the detection of mitochondrial DNA could be more difficult as the signal depends rather on the number of DNA breaks than on the DNA structure and therefore the signal manipulation to the benefit of the detection of the mitochondrial genome can be more difficult.
